# Improved Antisense Oligonucleotide Design to Suppress Aberrant *SMN2* Gene Transcript Processing: Towards a Treatment for Spinal Muscular Atrophy

**DOI:** 10.1371/journal.pone.0062114

**Published:** 2013-04-22

**Authors:** Chalermchai Mitrpant, Paul Porensky, Haiyan Zhou, Loren Price, Francesco Muntoni, Sue Fletcher, Steve D. Wilton, Arthur H. M. Burghes

**Affiliations:** 1 Centre for Neuromuscular and Neurological Disorders, The University of Western Australia, QE II Medical Centre, Perth, Western Australia, Australia; 2 Department of Biochemistry, Faculty of Medicine, Siriraj Hospital, Mahidol University, Bangkok, Thailand; 3 Department of Neurological Surgery, The Wexner Ohio State University Medical Center, Columbus, Ohio, United States of America; 4 Dubowitz Neuromuscular Centre, Institute of Child Health and Great Ormond Street Hospital, London, United Kingdom; 5 Department of Molecular and Cellular Biochemistry and Neurology, The Wexner Ohio State University Medical Center, Columbus, Ohio, United States of America; International Centre for Genetic Engineering and Biotechnology, Italy

## Abstract

Spinal muscular atrophy (SMA) is caused by loss of the Survival Motor Neuron 1 (*SMN1*) gene, resulting in reduced SMN protein. Humans possess the additional *SMN2* gene (or genes) that does produce low level of full length SMN, but cannot adequately compensate for loss of *SMN1* due to aberrant splicing. The majority of *SMN2* gene transcripts lack exon 7 and the resultant *SMN*Δ7 mRNA is translated into an unstable and non-functional protein. Splice intervention therapies to promote exon 7 retention and increase amounts of full-length *SMN2* transcript offer great potential as a treatment for SMA patients. Several splice silencing motifs in *SMN2* have been identified as potential targets for antisense oligonucleotide mediated splice modification. A strong splice silencer is located downstream of exon 7 in *SMN2* intron 7. Antisense oligonucleotides targeting this motif promoted *SMN2* exon 7 retention in the mature SMN2 transcripts, with increased SMN expression detected in SMA fibroblasts. We report here systematic optimisation of phosphorodiamidate morpholino oligonucleotides (PMO) that promote exon 7 retention to levels that rescued the phenotype in a severe mouse model of SMA after intracerebroventricular delivery. Furthermore, the PMO gives the longest survival reported to date after a single dosing by ICV.

## Introduction

Spinal muscular atrophy (SMA), the second most common autosomal recessive disorder in Caucasians, is the leading genetic cause of death in children under the age of 2 years [1]. Three forms of SMA are classified by the severity of the clinical presentation, with type I SMA being the most severe, and type III SMA the mildest. SMA is caused most commonly by loss of the *SMN1* gene, resulting in substantial reductions in levels of functional SMN protein. *SMN2*, which is retained in SMA, cannot adequately compensate for *SMN1* loss unless present at high copy number [2], [3]. The reason being that the majority of *SMN2* transcripts are inappropriately processed and lack exon 7 (SMNΔ7) [4]. The SMNΔ7 mRNA is translated into a truncated protein, which is incapable of self-association and leads to the production of a highly unstable SMN protein [5], [6]. Gene replacement therapy for SMN protein is one of the most direct approaches to treat SMA patients, however the delivery issue has long been an obstacle, because the blood brain barrier (BBB) restricts delivery of therapeutics to the central nervous system (CNS), where an insufficiency of functional SMN has the most severe consequences [7], [8], [9]. Subsequently, four groups have now produced scAAV9-SMN and reported a remarkable correction of the SMA phenotype with the highest titer virus resulting in mice surviving over 400 days [10], [11], [12], [13]. These studies demonstrated that replacement of SMN, at least early, is an effective therapy; however certainly difficulties remain in terms of sufficient vector production particularly if applied to older SMA patients. Interestingly, the scAAV9 does cross the BBB even in older animals in monkeys and can also give efficient transduction of motor neurons when introduced intrathecally, which reduces the amount of virus required [14].

An alternative approach to treat SMA is the application of antisense oligonucleotides to promote *SMN2* exon 7 retention. Relatively low expression of full-length SMN from *SMN2* arises from a C>T change in exon 7 that disrupts an exonic splice enhancer (SF2/ASF), with the concomitant creation of an exonic silencer element [15], [16]. This leads to suppression of exon 7 recognition and thus reduced incorporation of this exon in mature *SMN2* mRNA [4]. Critical intronic silencing motifs have been identified in introns 6 and 7 [17], [18], [19], the intronic silencing motif (ISS-N1) was identified as the most responsive target for the AO induced splice modification strategy [18], [20], [21].

This work describes further refinement and optimisation of phosphorodiamidate morpholino oligonucleotides (PMOs) targeting ISS-N1. A panel of 14 PMO of varying lengths was designed to anneal downstream of *SMN2* exon 7, encompassing the ISS-N1 region. Efficiency was studied *in vitro* in cultured fibroblasts from a type I SMA patient, and subsequently confirmed *in vivo* after the lead compound in a one timing dosing rescued a severely affected mouse model after intracerebroventricular (ICV) administration.

## Results

### Refining Oligonucleotide Design to Enhance SMN2 Exon 7 Incorporation

**Figure 1:**
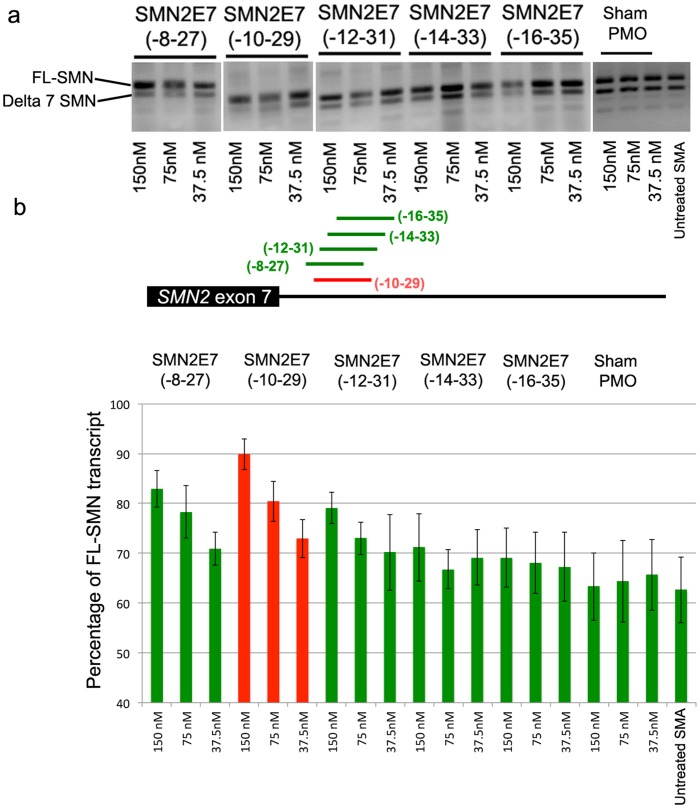
a) Splicing pattern of SMN transcripts in type I SMA patient cells transfected with a panel of 20-mer PMOs, unrelated negative control (Sham PMO) using lipofectin or untransfected fibroblasts (Untreated). b) The lower panel shows the average of percentage of *SMN2* exon 7 inclusion, as determined by densitometric analysis of RT-PCR gel images.

**Figure 2:**
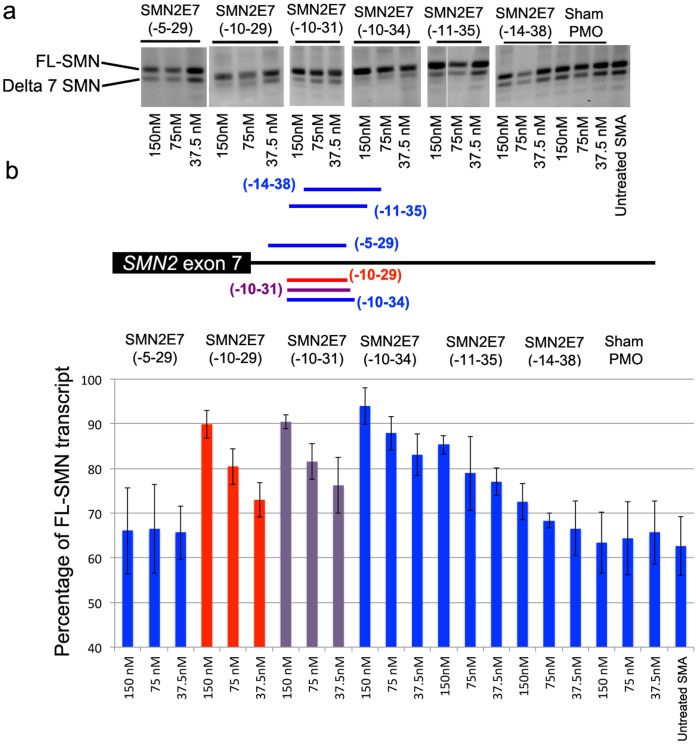
a) Splicing pattern of SMN transcript in type I SMA patient cells transfected with PMOs or unrelated negative control (Sham PMO) using lipofectin to compare the efficiency of PMOs of different length. b) The lower panel shows the average percentage of *SMN2* exon inclusion as determined by densitometric analysis of RT-PCR gel images.

**Figure 3:**
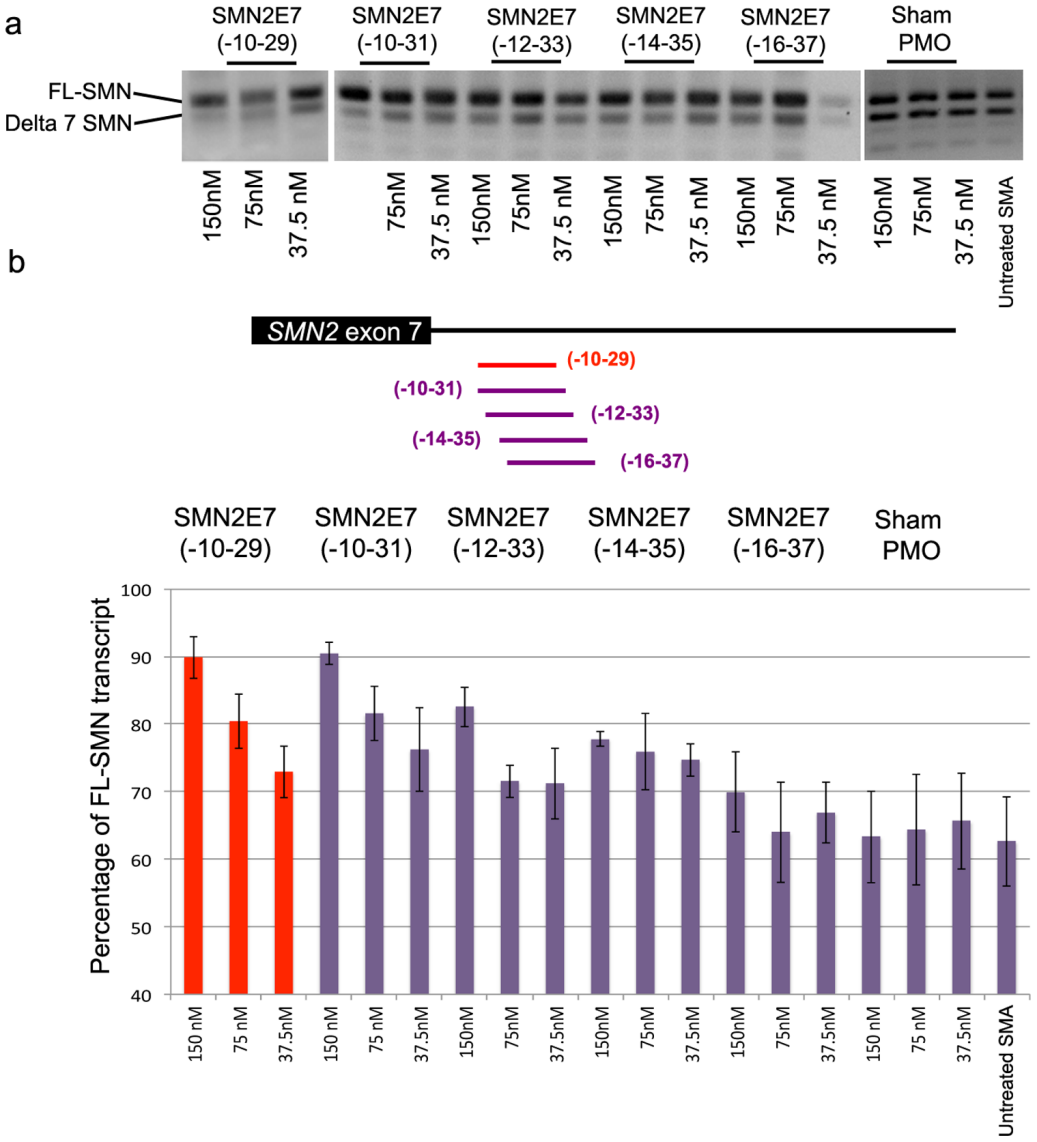
a) Splicing pattern of SMN transcript in type I SMA patient cells transfected with a panel of 22-mer PMOs or unrelated negative control (Sham PMO) using lipofectin. b) The lower panel shows the average percentage of *SMN2* exon inclusion as determined by densitometric analysis of RT-PCR gel images.

**Figure 4:**
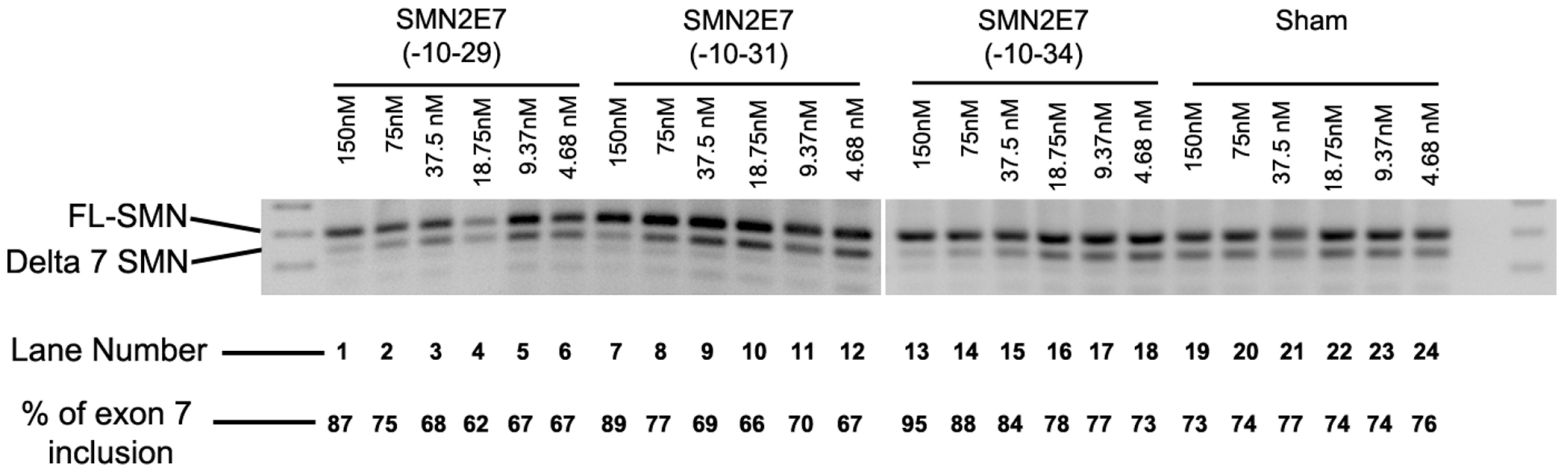
A titration study of the three lead PMOs: Splicing pattern of SMN transcript in type I SMA patient cells transfected with PMOs or unrelated negative control (Sham PMO) using lipofectin.at concentrations indicated. The percentage of *SMN2* exon inclusion as determined by densitometric analysis of RT-PCR gel images is also shown.

**Figure 5:**
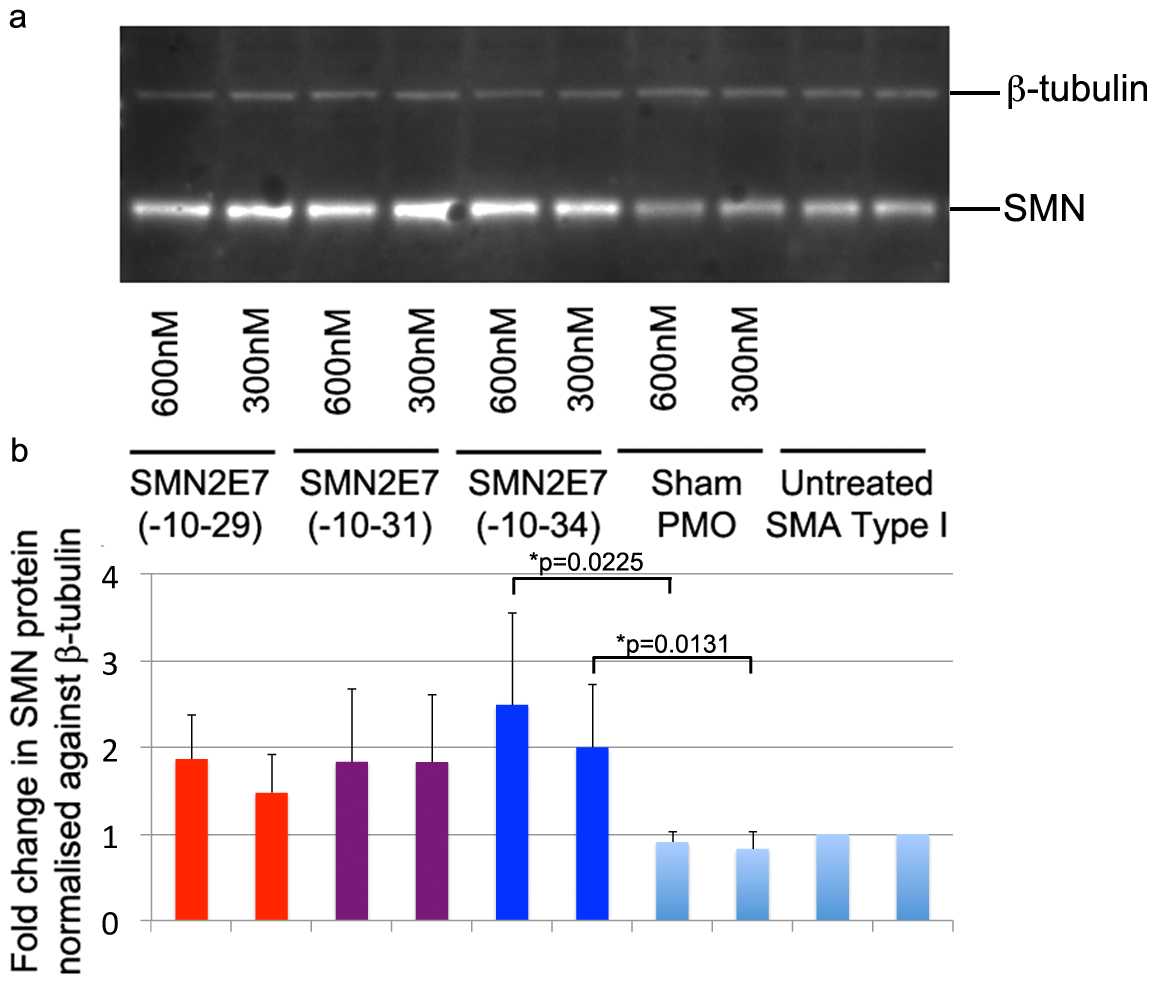
a) Western blot analysis of SMN and ß-tubulin in type I SMA patient cells transfected with PMOs or unrelated negative control (Sham PMO). b) The lower panel shows the fold change in SMN, normalised against ß-tubulin, as determined by densitometric analysis. P-values indicates statistical significance in SMN expression between PMO(-10-34) and sham PMO.

Efficiency of AO to promote FL-SMN can be enhanced by increasing the oligonucleotide length. Fourteen different PMOs varying in size of 20, 22 and 25 mers were designed to anneal to or near ISS-N1 (-10-25) **(** **Table 1** **and Figure S1)** and were transfected into fibroblasts derived from an SMA Type I patient using PMO:Leash with lipofection delivery system (**Figure 1** **,** **Figure 2** **,** **Figure 3** **,** **Figure 4** **,** **Figure 5**).

**Table 1:** The list of the 14 PMOs examined in this study.

Name	Sequence (5′–3′)	Size
SMN2E7D(-10-29)	AUU CAC UUU CAU AAU GCU GG	20
SMN2E7D(-10-34)	GUA AGA UUC ACU UUC AUA AUG CUG G	25
SMN2E7D(5-29)	AUU CAC UUU CAU AAU GCU GGC AGA A	25
SMN2E7D(-10-31)	AGA UUC ACU UUC AUA AUG CUG G	22
SMN2E7D(-12-33)	UAA GAU UCA CUU UCA UAA UGC U	22
SMN2E7D(-14-35)	AGU AAG AUU CAC UUU CAU AAU G	22
SMN2E7D(-16-37)	AAA GUA AGA UUC ACU UUC AUA A	22
SMN2E7D(-8-27)	UCA CUU UCA UAA UGC UGG CA	20
SMN2E7D(-12-31)	AGA UUC ACU UUC AUA AUG CU	20
SMN2E7D(-14-33)	UAA GAU UCA CUU UCA UAA UG	20
SMN2E7D(-16-35)	AGU AAG AUU CAC UUU CAU AA	20
SMN2E7D(-11-35)	AGU AAG AUU CAC UUU CAU AAU GCU G	25
SMN2E7D(-14-38)	AAA AGU AAG AUU CAC UUU CAU AAU G	25
Sham	CAG GAG ACG CCG GGC CUC CU	20

Of the five PMO20mers that annealed between intronic bases 8 to 35 downstream of exon 7, PMO(-10-29) was the most efficient at inducing the full length *SMN2* transcript retaining exon 7 (**Figure 1** **)**. Extending the length of PMO(-10-29) by five bases at the 5' end, PMO(-10-34), further improved the efficiency of exon inclusion in *SMN2*, clearly evident in a clear dose-dependant response. In contrast, extending PMO length by 5 bases at the 3' end, PMO(-5-29) resulted in diminished *SMN2* exon 7 inclusion level. Similarly, PMO25mers that targeted intron 7 regions further downstream of ISS-N1 (-14-38) also lost the ability to promote exon 7 retention in the *SMN2* transcripts (**Figure 2**). Further indication of the narrow window for PMO targeting to re-inforce *SMN2* exon 7 recognition and retention was seen after interrogating the area with a panel of PMO 22mers. PMO(-10-31) generated a clear dose dependant of exon 7 retention, whereas PMO(-12-33) was essentially inactive, as were other PMO22mers (PMO(-14-35) and PMO(-16-37)) (**Figure 3**).

### In vitro Titration Studies and Western Blotting

Titration studies of the three lead PMO candidates (20, 22, and 25mers) were undertaken to demonstrate dose responses, and identify the lead compound for further pre-clinical animal evaluation. Densitometric analysis of RT-PCR results showed that SMN exon 7 inclusion was induced by PMO(-10-34) at the concentration of 37.5 nM, while the 20 and 22mers PMOs were active only when transfected at higher concentrations (150 or 75 nM) in a type I SMA fibroblast (**Figure 4**).

Western blotting confirmed enhanced induction of the SMN protein in SMA type I fibroblasts. Transfection of SMA fibroblasts with PMO(-10-29) and PMO(-10-31) induced 1.86 and 1.83 fold increases in SMN protein at the concentration of 600 nM, while PMO(-10-34) treatment led to a 2.49 fold increase, when compared to the average of sham PMO treatment at equivalent concentrations and untreated SMA fibroblasts. At a transfection concentration of 300 nM, the fold change of induced SMN protein in SMA fibroblasts transfected with PMO(-10-29), PMO(-10-31) or PMO(-10-34) was 1.47, 1.83 and 2.00 fold, respectively (**Figure 5**).

**Figure 6:**
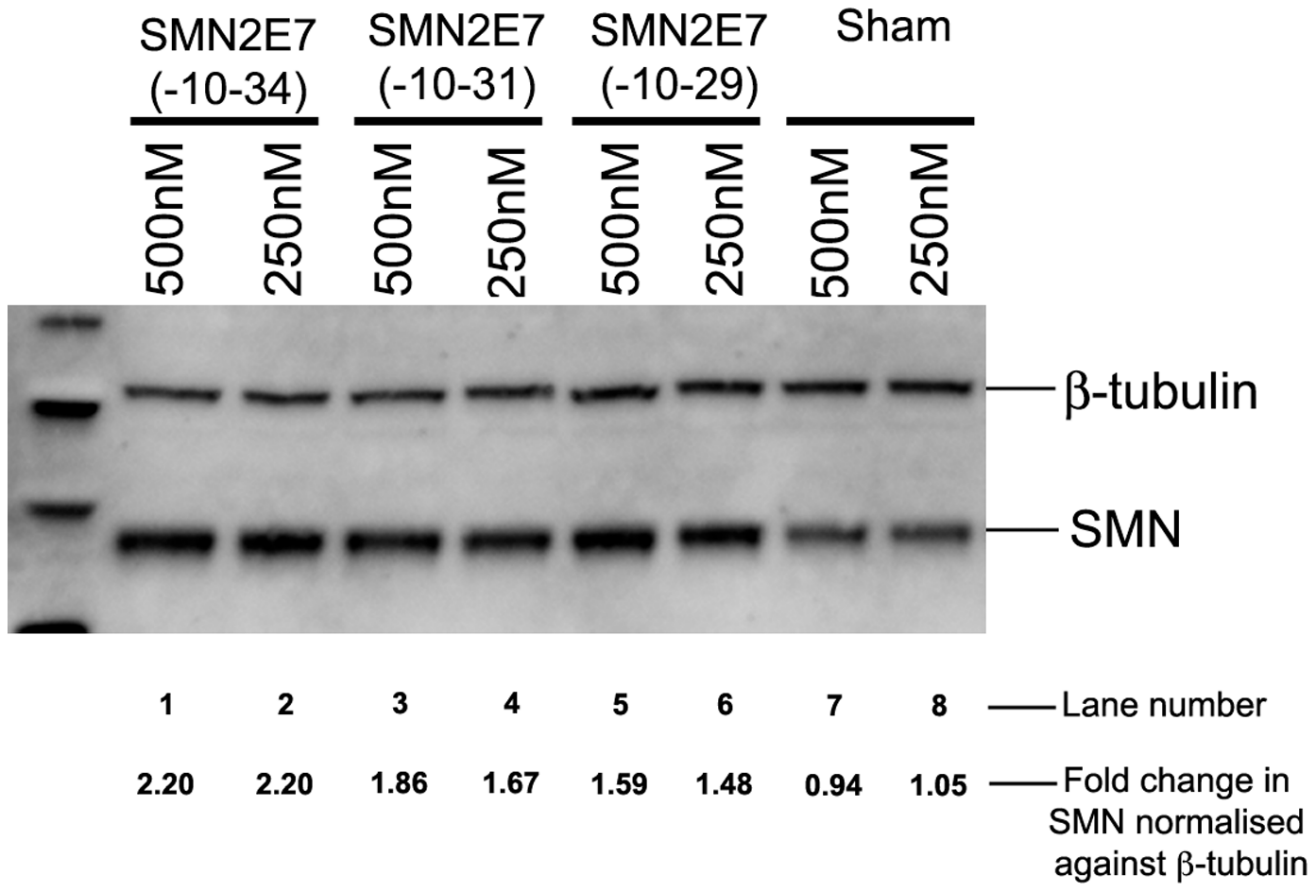
Western blot analysis of SMN in type I SMA patient fibroblasts nucleofected with PMOs or unrelated negative control (Sham PMO). The fold change in SMN expression, normalised against ß-tubulin is shown.

Subsequently, we utilised the second PMO delivery system to confirm the effect of selected PMO on the enhanced level of SMN protein expression in SMA fibroblast (**Figure 6**). Nucleofection was also evaluated to compare efficiency of PMO candidates. The level of induced SMN protein from SMA patient derived fibroblasts after nucleofection with PMO(-10-34) was higher than those of SMA fibroblasts treated with either PMO(-10-29) or PMO(-10-31) **(** **Figure 6** **)**. Taken together, PMO(-10-34) was identified as the most effective compound for induction of SMN protein after two different *in vitro* delivery systems and this oligonucleotide was selected for further *in vivo* evaluation.

### PMO(-10-34) Increased Expression of SMN Protein in SMA Carrier Mice

**Figure 7:**
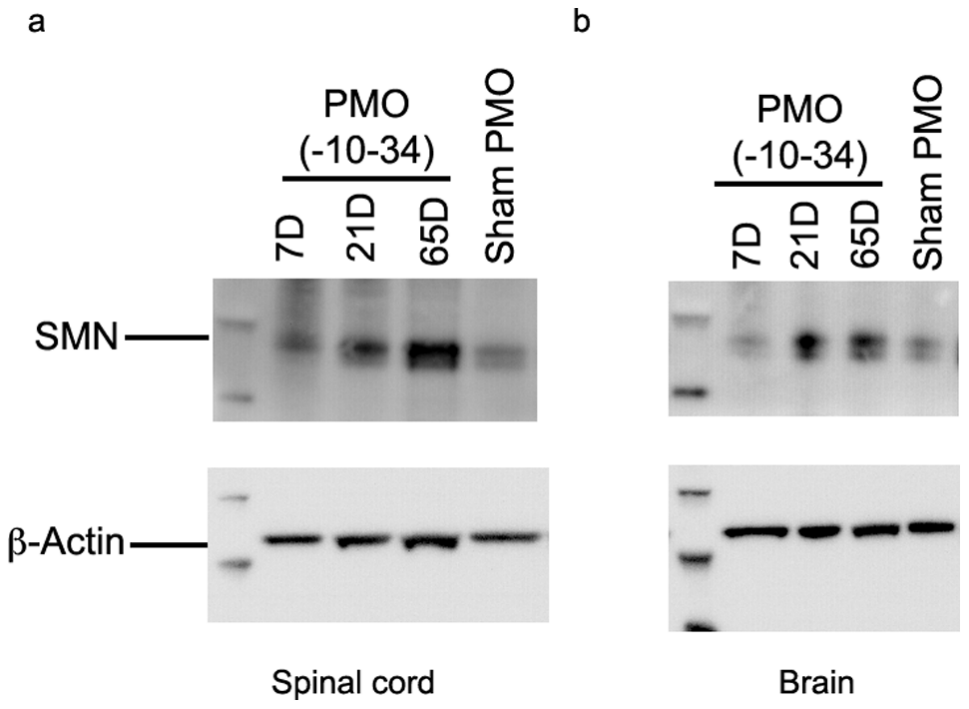
Western blot analysis of SMN and ß-actin in a) spinal cord and b) brain of SMA carrier mice (Smn^+/−^; SMN2^+/+^;Δ7^+/+^) intracerebroventricularly injected with 6 **mM of PMO(-10-34) or scrambled PMO control.** Tissues were collected at 7, 21 and 65 days after PMO(-10-34) injection and at 21 days after scramble PMO injection.

To study the effect of PMO on enhanced expression of SMN *in vivo*, SMA carrier mice (genotype Smn^+/−^; SMN2^+/+^; Δ7^+/+^) were used. PMO(-10-34) and scrambled PMO were intracerebroventricularly injected into SMA carrier mice. Human specific SMN antibody (KH antibody) was used for immunodetection to visualise enhanced human SMN protein expression. Substantially increased SMN protein expression in both spinal cord and brain was noted in 21 and 65 days after 6 mM injection of PMO(-10-34) (**Figure 7a and b**).

### PMO(-10-34) Extended Survival of a Severe Mouse Model of SMA

**Figure 8:**
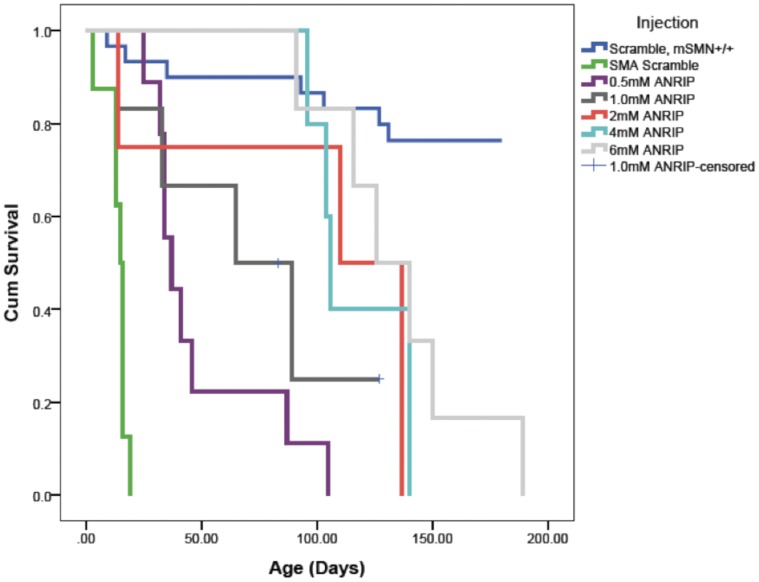
Survival curve of SMA mice intracerebroventricular injected with different dosage of PMO(-10-34).

**Figure 9:**
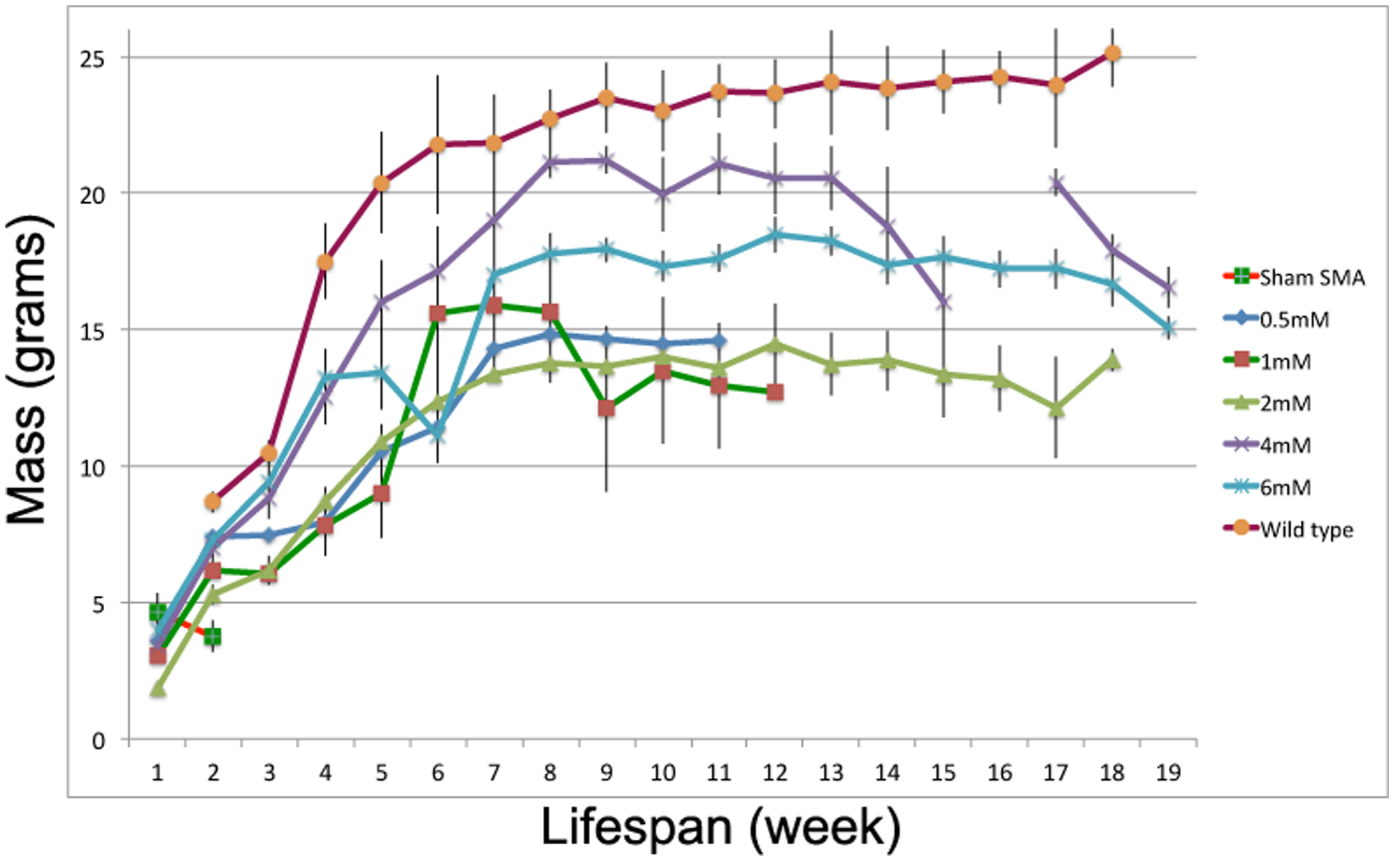
Mass of SMA mice intracerebroventricularly injected with PMO(-10-34) at five different doses.

Three different PMOs, PMO(-10-34), PMO(-10-29) and scrambled PMO were intracerebroventricularly administered into SMA mice (genotype *mSmn −/−; SMN2+/+;* Δ*7+/+)*, at doses indicated. The survival curve of SMA mice injected with a single ICV dose of PMO(-10-34) significantly prolonged survival of SMA mice at all doses (**Figure 8** **and** **Table 2**). Median survival increased from 15 days (untreated SMA animal, scrambled PMO injection) to a median range of 37 to 126 days for the five dosing strategies. Among SMA mice treated with five different doses of PMO(-10-34), pairwise analysis indicated that the median survival of SMA mice injected with 0.5 mM was significantly different from those mice treated with either 2, 4 or 6 mM. In keeping with the trend of the survival, the weight of mice injected with PMO(-10-34) at 4 or 6 mM was higher than that of mice injected with either 0.5, 1 or 2 mM of PMO(-10-34) (**Figure 9**).

**Table 2:** Comparison of mean and median survival time of SMN mice injected with PMO(-10-34) or Scramble.

Doses	Mean survival time (day)	Median survival time (day)	Log Rank test P-value
2 mM	**99.5** VS 83.0	**110** VS 83	0.543
4 mM	**117.2** VS 111.0	**110** VS 106	0.700
6 mM	**135.3** VS 102.9	**126** VS 112	0.097

Median survival of SMA mice injected with PMO(-10-34) and PMO(-10-29) at dosages of 2, 4 and 6 mM were compared, and the median survival time of PMO(-10-34) treatment group was higher than that of mice treated with PMO(-10-29) at all three doses (**Table 3**). However, these differences were not statistically significant (P-value 0.084 for median survival time, P-value 0.097 for Log Rank test for survival curve).

**Table 3:** Comparison of the mean and median survival time between the mice injected with different dosages of PMO(-10-34) or PMO(-10-29).

Dosage	Mean survivaltime (day)	95% CI	Median survivaltime (day)	95% CI	Log-Rank testP-Value
0.5 mM	49.0	(30.9, 67.01)	37	(28.2, 45.8)	<0.001*
1.0 mM	72.7	(39.3, 106.0)	65	(11.2, 118.8)	0.005*
2 mM	99.5	(29.2, 42.3)	110	(29.6, 190.4)	0.03*
4 mM	117.2	(98.7, 135.7)	106	(101.7, 110.3)	0.001*
6 mM	135.3	(108.7, 162.0)	126	(97.2, 154.8)	<0.001*

* indicate statistical significant.

## Discussion

We previously reported that increasing oligonucleotide length could enhance the efficiency of some compounds, at least when targeting some exons in the dystrophin gene transcript [22]. We have employed a similar strategy to optimise *SMN2* pre-mRNA processing with a systematic refinement of oligonucleotide design. The initial microwalk was undertaken by using five 20mer PMOs, which annealed to the intron 7 between intronic bases 8 to 35 base downstream from exon 7, and only PMO(-10-29) and PMO(-8-27) increased levels of full-length SMN (FL-SMN) transcript above that observed in untreated cells. PMOs extended by 5 bases at either end of PMO(-10-29) were evaluated, and the 25mer PMO (PMO(-10-34)) was found to be most effective in promoting exon 7 inclusion. This was most evident after *in vitro* transfection into SMA patient fibroblasts at the lower concentrations, while 22-mer PMO (PMO(-10-31)) induced less pronounced levels of the FL-SMN transcript.

We have previously shown that subtle changes in oligonucleotide design can significantly influence the efficiency of AO, with respect to exon skipping in the dystrophin gene [23], [24], and this study confirms that minor changes in oligonucleotide sequences can be most significant when targeting splice silencers. PMO(-11-35), displaced by only one base further into the intron than PMO(-10-34), showed almost no SMN exon 7 inclusion activity after transfection of PMO:leash lipoplexes at the concentrations of 75 and 37.5 nM. Four overlapping PMO22mers were designed and evaluated to further refine optimal coordinates to promote the FL-SMN transcript. PMO(-10-31) also induced pronounced exon 7 inclusion, but once again a narrow window of oligonucleotide targeting was observed. This finding was in keeping with the evolutionary significance of the ISS-N1 motif in humans, as mouse Smn gene does not contain the first few bases of ISS-N1 motif [19].

Titration studies were undertaken to rank SMN2 exon 7 retention efficiency of PMO(-10-29), PMO(-10-31) and PMO(-10-34) in type I SMA fibroblasts. PMO(-10-34) induced inclusion of SMN exon 7 at all concentrations evaluated, while PMO(-10-29) and PMO(-10-31) only increased FL-SMN transcript levels after transfection at two highest tested concentrations (150 and 75 nM), as assessed by RT-PCR assays. These PMOs were further evaluated by comparing their efficiencies at inducing SMN protein in type I SMA fibroblasts using different protocols (leash and cationic lipoplex or nucleofection). PMO(-10-34) induced substantial more SMN expression than the other two shorter PMOs, based upon western blotting. The finding is in keeping with another study to compare three oligonucleotides targeting ISS-N1 [25].

Subsequently, PMO(-10-34) was assessed for its effect on enhanced SMN protein expression in SMA carrier mice using a human specific antibody. Robust expression of SMN protein observed at day 21 and 65 after single ICV injection confirmed our observation *in vitro* and this compound was chosen as the lead candidate for further studies and evaluated in a severe mouse model of SMA, allowing a direct comparison of our previously reported PMO(-10-29) [21]. Untreated, the severely affected pups normally succumb to the disease by postnatal day 15, and they can survive a median of 2–3 days longer, if maternal diet contains higher fat content [26]. In line with our earlier studies, a single ICV injection of PMO(-10-34) significantly prolonged the lifespan of SMA mice, compared to sham PMO, at all 5 tested doses.

Average survival of SMA mice injected with PMO(-10-34) was not statistically significant between the three dose cohorts, i.e. 2 mM, 4 mM and 6 mM, and was similar to that previously reported on SMA mice injected with PMO(-10-29) [21]. SMA mice were treated with lower dosages to identify minimum thresholds for therapeutic doses. It was found that the survival of the SMA mice, injected with PMO(-10-34) at 0.5 mM, was significantly shorter than littermates injected with either 2 mM, 4 mM or 6 mM of PMO(-10-34). However, when considering the mass of injected mice, we observed that mice injected with either 4 mM or 6 mM of PMO(-10-34) gained substantially more weight compared to that of mice injected with other lower doses. We inferred that the therapeutic doses for SMA mice should be 4–6 mM considering both survival and the weight gain. This study demonstrated the optimal dose for single bolus ICV injection; however a number of delivery methods; such as continuous infusion through implantable intrathecal or intraventricular catheter or multi-dose bolus injection, are also options for drug delivery in human. A study interrogating the method of PMO delivery is required in particular a redosing regime to determine whether further survival benefit can be obtained. Indeed the comparison of ASO needs to consider both the dose and frequency of redosing. When comparing PMO(-10-29) to PMO(-10-34) the effective cost and redosing regime are all critical in determining what moves forward for clinical experimentation.

There were no obvious adverse effects on the SMA mice injected with PMO(-10-34) in their first 50 days of life. However, these mice displayed ear and tail necrosis, arising from day 50 through 90, similar to that observed in PMO(-10-29) treated SMA mice [21]. Some PMO(-10-34) treated animals developed paw or snout necrosis, but this was only observed in SMA mice that lived generally more than 100 days. Some of the older survivors developed bowel and bladder obstructions, and required manual decompression and systemic antibiotics to alleviate the symptoms. In addition to bladder and bowel obstruction, priapism and peripheral necrosis were also observed in the longer lived SMA mice, and these findings were what previously observed in rescued SMA mice [21], [27]. One key in the future is whether redosing the ASO in an effective manner so it alters SMN2 splicing distal to the inoculation site reduces or eliminates these issues. The digital necrosis was also previously reported in two patients with type I SMA who received proactive nutritional and respiratory care and therefore lived longer [28]. These morbidities could represent dysfunction of the autonomic nervous system (ANS), which is partially located outside the central nervous system. Hua *et al.* demonstrated that additional delivery of oligonucleotide either by ICV injection at postnatal day 1 or by subcutaneous injection at postnatal day 5 or 7 can delay necrosis [29]. However the overall dose of the oligonucleotide was higher and at the early stage of mouse development (P0–P7) the blood brain barrier is open to AO treatment making it hard to determine whether this is due to a peripheral or central targets. Taken together, re-administration of PMO could rescue the peripheral necrosis and some or all of the peripheral structure of ANS may perhaps need to be treated, as it has also demonstrated using systemic administration of vivo-PMO, which is more effective than PMO to target the SMN gene in the periphery [25]. This study assessed the consequences of a single administration of PMO, and Longer term survival with reduced complications observed in SMA mice or type I SMA patients will obviously require appropriate re-administration protocol to deliver PMO. In this regard, it will be important to obtain adequate distribution of the PMO in the CNS in both neonatal and adult animals.

Comparative analysis of the two PMOs on the survival of SMA mice indicated that PMO(-10-34) significantly extended the life-span of treated SMA mice, and the effectiveness is dose responsive. When comparing to PMO(-10-29), the survival of mice injected with PMO(-10-34) is equal to, or higher than mice injected with PMO(-10-29), even though the difference do not reach statistical significance. However, the differences of the survival between two treatment groups is more prominent at higher dose. The key question will be the dosing and redosing scheme that is optimal for long-term correction of SMA mice.

In summary, we have undertaken a systemic screening of oligonucleotides targeting ISS-N1 and substantially enhanced oligonucleotide design, so that the lead compound PMO(-10-34) increased FL-transcript and SMN protein *in vitro* and prolong the survival *in vivo*. While there was no statistical difference between the shorter PMO(-10-29) when compared to the longer PMO(-10-34), there was a trend to longer survival. The question that occurs is what happens on multiple injections; a critical question that has not been addressed for either the morpholino or MOE chemistries. We have previously re-administered the PMO(-10-29) at postnatal 28 days after ICV injection at P0 and additional injection did not further prolong the survival. However we did not test distribution of the PMO in the CNS of adult animals and this is critical to the effectiveness of therapy. We suggested that in the current study the PMO(-10-34) exerts an affect on *SMN2* that lasts slightly longer than a PMO(-10-29). If this is the case then the difference in use of PMO(-10-29) and PMO(-10-34) may well be the frequency of reintroduction. Currently we would predict an approximately a monthly introduction of AO based on the decay of SMN amounts [21]. Further studies are required to determine the optimal reintroduction strategy in SMA, a critical parameter in the successful implementation of antisense oligonucleotides in SMA.

## Materials and Methods

### Cell Culture Propagation

A type I SMA fibroblast obtained from Coriell Cell Repositories was propagated in 10 ml of 10% foetal bovine serum in Dulbecco minimum eagle media (DMEM) with Glutamine supplemented with penicillin (10 U/ml) and streptomycin (10 µg/ml). 1.5×10^4^ cells were seeded into 24-well and subsequently incubated for 24 hours prior to transfection.

### Phosphorodiamidate Morpholino Oligonucleotide and Primers

Oligonucleotide nomenclature specifies the intronic annealing coordinates, hence PMO(-10-29) describes a compound annealing between bases 10 and 29 from the beginning of *SMN2* intron 7. PMOs were manufactured and supplied by Gene-Tools (Philomath, USA). The sequences of the PMO investigated are designed to refine the silencing motif (ISS-N1) previously described [19], and the list of tested PMO was provided in Table 1. For *in vitro* experiment, PMOs were resuspended in normal saline for fibroblast transfection. The DNA leashes for PMO transfection and the primers for RT-PCR were synthesized by Geneworks (Adelaide, Australia). For the ICV injection, PMOs were prepared in sterile 0.9% sodium chloride, and mixed with Evans Blue (final concentration 0.04%); three different molar concentrations were prepared at different concentrations and stored at −20°C.

### PMO Transfection

PMOs:DNA leash lipoplex method was used to enhance transfection efficiency, as previously reported. [30] Briefly, the initially PMO:leash working solutions were prepared at a final concentration of 50 µM of both PMO and respective leash at a 1∶1 molar ratio. After annealing, this PMO mixture was then utilised to transfect onto a type I SMA fibroblasts, using lipofectin agent (Lifetechnology) at a ratio of 2∶1 Lipofectin to PMO mix. Lipofectin was mixed with DMEM (Invitrogen) to a final volume of 100 µl. The PMO mix, which had been diluted to 100 µl in DMEM, was then combined with the Lipofectin/DMEM mixture. The mixture was then made up to a final volume of 1 ml using antibiotic-free medium. 500 µl aliquots of this mixture were added to each well of 24-well plate containing SMA fibroblast and incubated for 48 hours.

Nucleofection was undertaken using Amaxa 4D kit (Lonza). Two microliter of stock PMO was added into a cuvette, and a quarter of a million of SMA fibroblasts were resuspended into 18 µl of pre-warmed transfection solution, and then add to the cuvette. The mixture of fibroblast solution and PMO was subsequently nucleofected using Amaxa 4D Machine.

### RNA Extraction and RT-PCR

Total RNA was harvested from type I SMA fibroblast using Trizol (LifeTechnology), according to the manufacturer's protocol. One step RT-PCR using Superscript III: ∼150 ng of total RNA was used as a template and incubated for 30 min at 55°C, and at 94°C for 2 min, before 28 rounds of 94°C for 40 sec, 56°C for 1 min and 68°C for 1 min using exon 4F and 8R primers. PCR products were fractionated on 2% agarose gels in Tris-Acetate-EDTA buffer and the images captured on a Chemismart-3000 gel documentation system and analysed with Bio1D software to quantitate band weight and estimate ratios of SMN-FL and SMNΔ7. Product identity was confirmed by band purification and DNA sequencing as necessary.

### Western Blotting

Protein extracts were prepared according to Cooper *et al*., 2003, [31] but with 15% SDS. SDS-PAGE electrophoresis was performed using NuPAGE Novex 4–12% BIS/Tris gels run at 200V for 55 minutes. Proteins were transferred to Pall Fluorotrans W PVDF membranes at 30 V for 1 hour at 18°C. For SMN detection in fibroblasts, MANSMA1 antibody was applied at 1∶100 dilution overnight at 4°C. For SMN detection in Smn mice, human specific SMN antibody (KH antibody) was applied at 1∶20 dilution overnight at 4°C [32]. Invitrogen Western Breeze kit was used for immunodetection. Quantification was performed on a Vilber Lourmat Chemi-Smart 3000 system using Chemi-Cap software for image acquisition and Bio-1D software for image analysis. ß-tubulin was detected by a mouse monoclonal antibody (BD Pharmingen, Cat. no 556321), as a reference loading protein. ß-actin was detected by a rabbit monoclonal antibody (Cell Signaling, Cat. no 4970), as a reference loading protein for mouse tissue.

### Generation of SMA Mice and Mouse Genotyping

Δ7 carrier breeding mice (*SMN2+/+; Smn+/−; SMNΔ7+/+*) were crossed to generate three types of offspring varying in the mouse Smn genotype: *Smn +/+*, *Smn +/−*, *Smn −/−* as previously described. All breeding and subsequent use of animals in this study were approved by the IACUC of The Ohio State University, Columbus, Ohio. SMA mouse were genotyped at postnatal day 0 via tail clip, and all genotyping was performed during day 0 as described previously [11].

### Intracerebroventricular Injection of SMA Mice

ICV injection was performed as previously described [21].

### Statistical Analysis

T-test was used to compare the fold changes in SMN expression between PMO(-10-34) transfected and sham PMO treated samples. For the survival curve analysis, data are expressed as means plus standard errors. Kaplan–Meier curves were generated from the survival data and tested using the Mantel–Cox log-rank test. All statistical analyses were performed using SPSS, v.16.0.

## Supporting Information

Figure S1 **Schematic diagram representing PMO binding coordinates on human** ***SMN2*** **intron 7.**
(PDF)
