# Supplementary figures and images for: Improved Antisense Oligonucleotide Design to Suppress Aberrant SMN2 Gene Transcript Processing: Towards a Treatment for Spinal Muscular Atrophy
Source: PLoS One. 2013 Apr 22;8(4):e62114. doi: 10.1371/journal.pone.0062114 (PMC3632594; doi:10.1371/journal.pone.0062114)

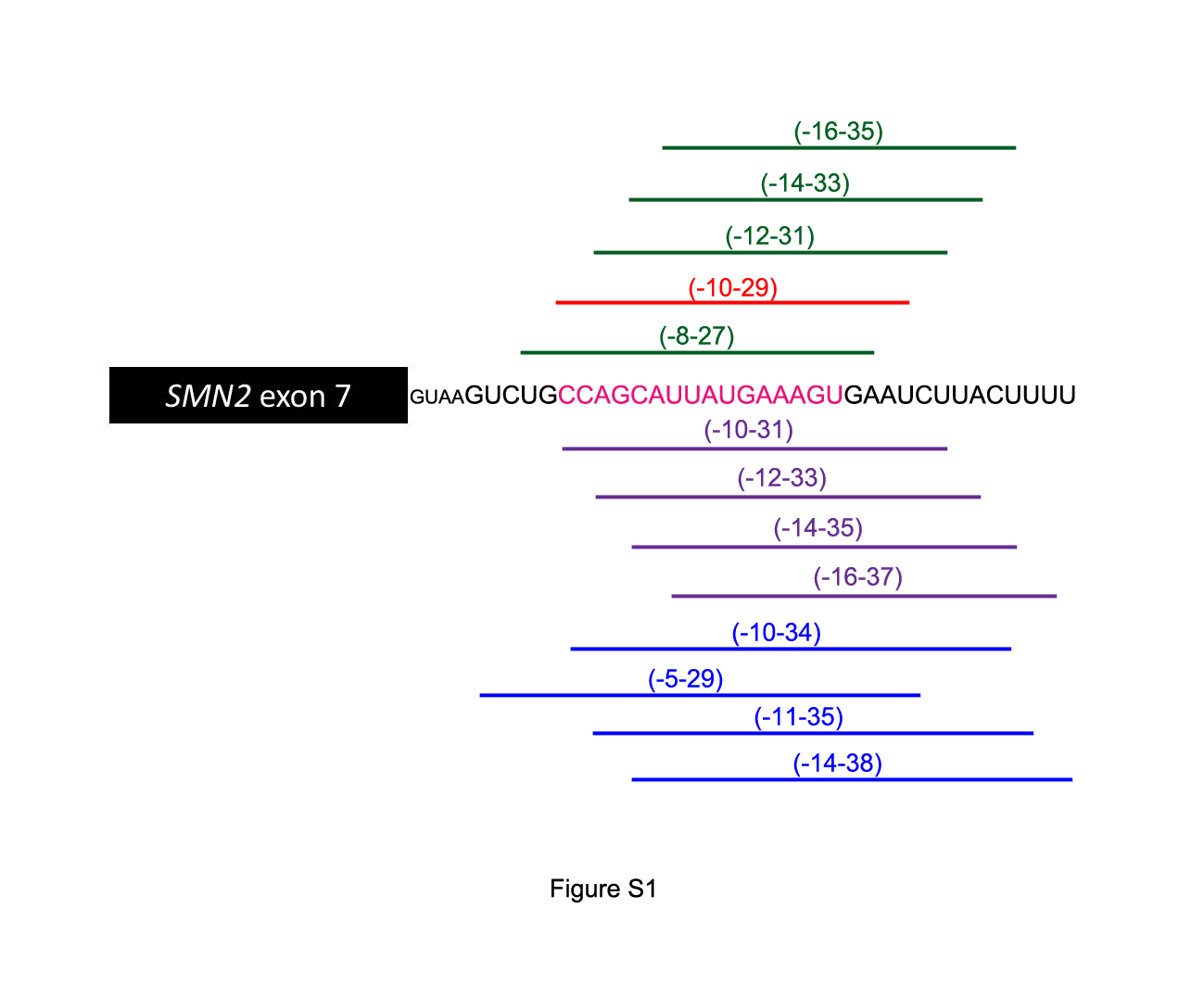

Supplement: Figure S1 — Schematic diagram representing PMO binding coordinates on human SMN2 intron 7. (PDF) [file pone.0062114.s001.pdf]
